# Insight into *Nephrocan* Function in Mouse Endoderm Patterning

**DOI:** 10.3390/ijms21010008

**Published:** 2019-12-18

**Authors:** Martina Addeo, Silvia Buonaiuto, Ilaria Guerriero, Elena Amendola, Feliciano Visconte, Antonio Marino, Maria Teresa De Angelis, Filomena Russo, Luca Roberto, Pina Marotta, Nicola Antonino Russo, Anna Iervolino, Federica Amodio, Mario De Felice, Valeria Lucci, Geppino Falco

**Affiliations:** 1 Istituto di Ricerche Genetiche “G. Salvatore”, Biogem s.c.ar.l, Ariano Irpino, 83031 Avellino, Italy; martina.addeo@gmail.com (M.A.); ilaria.guerriero.ig@gmail.com (I.G.); mariateresadeangelis211285@gmail.com (M.T.D.A.); filomena_russo1982@libero.it (F.R.); luca.roberto@biogem.it (L.R.); russonico@gmail.com (N.A.R.); pinamarotta82@gmail.com (P.M.); amodio.federica@yahoo.it (F.A.); anna.iervolino@biogem.it (A.I.); 2 Dipartimento di Biologia, Università degli Studi di Napoli “Federico II”, 80126 Napoli, Italy; buonaiutosilvia@gmail.com (S.B.); antoniomarmail@gmail.com (A.M.); elena.amendola@unina.it (E.A.); 3 Istituto per l’Endocrinologia e l’Oncologia Sperimentale “G. Salvatore”, CNR, 80131 Napoli, Italy; mario.defelice@unina.it; 4 CEINGE Biotecnologie Avanzate s.c.a.r.l., 80131 Napoli, Italy; visconte@ceinge.unina.it

**Keywords:** *Nephrocan* gene, transcriptional variants, embryonic stem cells, differentiation, definitive endoderm, (CRISPR)/CRISPR-associated systems 9 (Cas9), mouse model

## Abstract

Endoderm-derived organs as liver and pancreas are potential targets for regenerative therapies, and thus, there is great interest in understanding the pathways that regulate the induction and specification of this germ layer. Currently, the knowledge of molecular mechanisms that guide the in vivo endoderm specification is restricted by the lack of early endoderm specific markers. *Nephrocan* (*Nepn*) is a gene whose expression characterizes the early stages of murine endoderm specification (E7.5–11.5) and encodes a secreted N-glycosylated protein. In the present study, we report the identification of a new transcript variant that is generated through alternative splicing. The new variant was found to have differential and tissue specific expression in the adult mouse. In order to better understand *Nepn* role during endoderm specification, we generated *Nepn* knock-out (KO) mice. *Nepn*^−/−^ mice were born at Mendelian ratios and displayed no evident phenotype compared to WT mice. In addition, we produced nullizygous mouse embryonic stem cell (mESC) line lacking *Nepn* by applying (CRISPR)/CRISPR-associated systems 9 (Cas9) and employed a differentiation protocol toward endoderm lineage. Our in vitro results revealed that *Nepn* loss affects the endoderm differentiation impairing the expression of posterior foregut-associated markers.

## 1. Introduction

The molecular mechanisms controlling the early cell fate decisions in mammals is of major interest to regenerative medicine, since guided differentiation of embryonic stem cells (ESCs) toward a specific lineage is an appealing opportunity to provide bases for exploring new therapies. The definitive endoderm (DE), one of the three primary germ layers formed during gastrulation, gives rise to the gastrointestinal organs, such as stomach, pancreas, liver, and intestine, which are of great importance in their therapeutic aspects [1,2]. In mouse, endoderm, together with mesoderm and ectoderm, is formed from the epiblast through the process of gastrulation beginning at approximately day 6.5 of gestation [3]. As gastrulation initiates, the pluripotent epiblast cells are recruited to a structure known as the primitive streak (PS), where they undergo an epithelial-to-mesenchymal transition (EMT) giving rise to mesoderm and definitive endoderm [4,5]. The endodermal lineage acquires an epithelial fate and intercalates into the overlying visceral endoderm (VE) to form the DE [6,7,8]. The formation of DE involves a migration from mesenchymal PS to an epithelial endoderm layer; therefore, it is commonly regarded as an mesenchymal-to-epithelial transition (MET) process [9]. Once the primary germ layers are established, the endoderm undergoes a complex series of changes to give rise to a primitive gut tube [10]. The derivative organs of the definitive endoderm begin to develop, arising as small buds in the epithelial layer that then further differentiate as embryogenesis proceeds [2]. Many studies have shown that members of the Transforming Growth Factor-β (TGF-β) superfamily, including Nodal, Activins, and Bone Morphogenetic Proteins (BMPs), as well as their inhibitors, such as the Forkhead domain factors and Sox17 HMG domain proteins, are responsible for the induction and specification of DE lineage [11,12,13,14]. Nevertheless, the knowledge of genetic mechanisms that guide the in vivo endoderm specification is restricted by the lack of early endoderm specific markers. In order to further improve the comprehension of pancreatic cellular ontogeny, we performed a global expression profile of dorsal pancreatic bud at E 10.5 and *Nepn* was identified among the most enriched genes [15]. *Nepn* encodes a small leucine-rich repeat protein (SLRP) [16] that can act as a secreted inhibitor of TGF-β signaling [17], a key regulator in endoderm development. *Nepn* is expressed in the definitive endoderm that emerges during gastrulation, as well as its derivative the gut tube epithelium and the early pancreas primordium (E7.5−11.5) [18]. In adult mice, *Nepn* is expressed mainly in the kidney [17]. In the present study, we report a novel transcript variant of the gene that arises as a result of alternative splicing and is expressed in tissue and developmental stage specific manner. The newly identified variant was altogether absent between the embryonic stages E8.5-E11.5 and was found to be expressed differentially across different adult tissues. Moreover, we describe in vivo and in vitro experiment examining *Nepn* involvement in posterior foregut development. We generated and analyzed constitutive *Nepn*^KOfirst^ mice (called *Nepn*^−/−^ or *Nepn*^+/−^). *Nepn*^−/−^ mutants were viable and fertile, exhibited normal body weight and displayed normal glucose tolerance and renal functionality. By using the CRISPR/Cas9 technique [19], we also generated the *Nepn* deficient mESCs. We programmed differentiation of wild type and mutant mESCs toward the endoderm lineage and demonstrated that the *Nepn* CRISPR-knockout impairs the expression of posterior foregut specific markers such as *Gata4/6* and *Sox9*. These experiments will improve the knowledge of mechanisms that guide the in vivo endoderm development, which is an important goal to sustain the progression of new therapeutic strategies to be used in the treatment of disease involving endoderm derived organs.

## 2. Results

### 2.1. Evidence for a New Transcript Variant of Nephrocan in mouse

**Figure 1:**
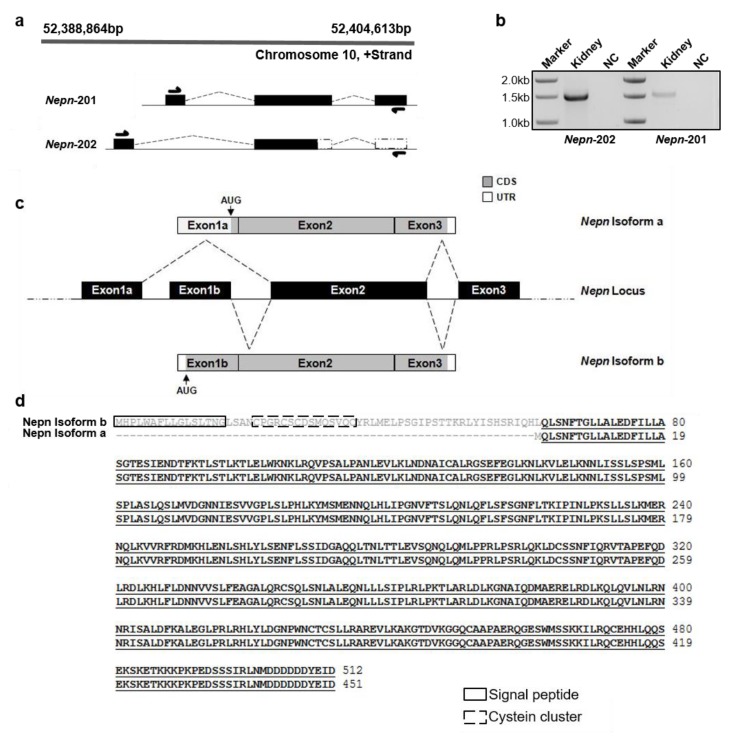
Evidence for a new exon of *Nephrocan* in mouse. (**a**) The sequence of *Nepn* gene in GenBank runs from 52,388,864 bp to 52,404,613 bp on chromosome 10 (top). Schematic of transcripts reported on Ensemble database and primer designing for RT-PCR (bottom). (**b**) Mouse kidney tissue was used to study the expression of the novel transcript by RT-PCR. NC, negative control with no DNA template. (**c**) Schematic representation of the genomic organization of mouse *Nepn* leading to alternative splicing. Rectangular boxes refer to exons (size of boxes are indicative of the relative size of exons) and interconnecting lines as introns. The dashed puckered lines show the splicing pattern of the exons. The novel transcript, named *Nepn* isoform a, is formed by splicing of Exon1a with Exon2 skipping Exon1b. The translation initiation site “AUG” is indicated with a downward arrow in the new transcript. (**d**) Alignment of Nepn isoform b (upper sequence) and isoform a (lower sequence) obtained using the Sequence Manipulation Suite programs. Full blot is showed in Supplementary Information.

*Nepn* gene is localized on murine chromosome 10 (10qB3) on positive strand, spanning 12,997 bases from position 52,388,864 bp to 52,404,613 bp (Figure 1a top). To date, only one transcript of 1826 bp (NM_025684.2 indicated as *Nepn*-201) has been validated, which contains three exons and codes for a protein containing 512 amino acids [17]. Another incomplete transcript is reported on Ensembl database (Transcript ID: ENSMUST00000219730.1 indicated as *Nepn*-202) consisting of a different exon 1 and a portion of exon 2 with a predicted 201 amino acids polypeptide. Based on this evidence, we hypothesized the presence of a second isoform composed by an alternative exon 1, exon 2 and exon 3. To experimentally validate the existence of this new transcript, we designed two forward primers which recognize specifically the two putative alternative exons 1, and a reverse primer located in exon 3 (Figure 1a bottom). The RT-PCR performed on adult mouse kidney revealed an expected band of 1536 bp for *Nepn*-201 (hereafter *Nepn* isoform b), and another smaller transcript of 1496 bp for *Nepn*-202 (hereafter *Nepn* isoform a), indicating that both full-length isoforms exist and are detectable (Figure 1b). As shown in the schematic illustration of *Nepn* genomic locus (Figure 1c), *Nepn* isoform a (1863 bp) transcript consists of three exons: exon 1a, exon 2 and exon 3. Almost whole exon 1a consists of a 5′ untranslated region (UTR) except for the last two bases, which form the start codon (AUG) with the first base of the exon 2. The open reading frame of *Nepn* isoform a encodes a polypeptide of 451 amino acids, which differs from Nepn isoform b protein for the absence of 62 amino acids at the N-terminus and the consequent loss of a putative signal peptide that directs the protein secretion and cysteine cluster (Figure 1d).

### 2.2. Nepn Isoforms Expression Pattern in Mouse Development and Tissues

**Figure 2:**
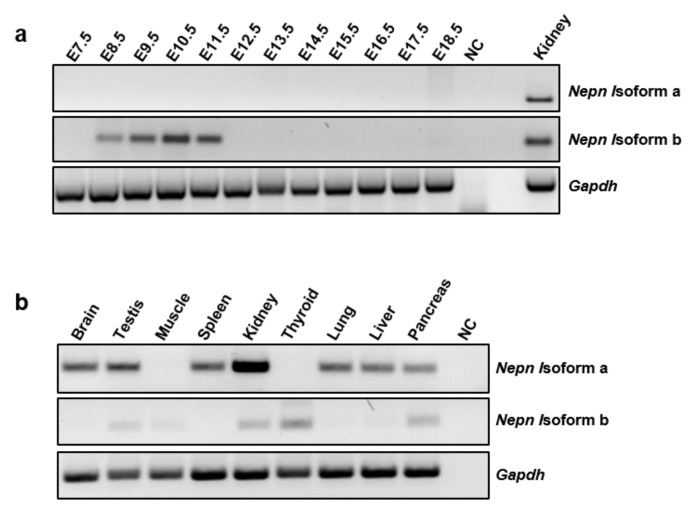
Expression pattern of *Nepn* isoforms. *Nepn* isoforms a and b were amplified by RT-PCR on total RNA from adult mouse organs and embryos homogenate. Two different PCR reactions were performed on the same template cDNA using specific oligos. (**a**) *Nepn* isoforms a and b expression in mouse embryo development; (**b**) *Nepn* isoforms a and b expression in adult mouse tissues. *Gapdh* amplification was performed as a control. Full blot is showed in Supplementary Information.

As the existence of a new *Nepn* transcript has been validated, we analyzed the expression pattern during embryogenesis and the tissue specific distribution of both isoforms. Interestingly, during embryo development, only *Nepn* isoform b transcript is detectable, and in particular, it shows a well-defined time window expression between the embryonic stages E8.5–E11.5 (Figure 2a). Since the restricted expression to the dorsal pancreatic bud (DPB) at the developmental stage E10.5 has been already reported [17], this strengthens the hypothesis that it could play an important role in pancreatic specification. We also analyzed the expression of both isoforms in several adult mouse tissues, deriving from all three germ layers. As shown in Figure 2b, the expression of *Nepn* isoform a was detected in most of the tissues analyzed, except for muscle and thyroid, whereas *Nepn* isoform b was found to be expressed in kidney, thyroid, pancreas and to a much lesser extent, in testis and muscle.

### 2.3. Nepn Knockout First Mouse (Nepn^KOfirst^) Generation

**Figure 3:**
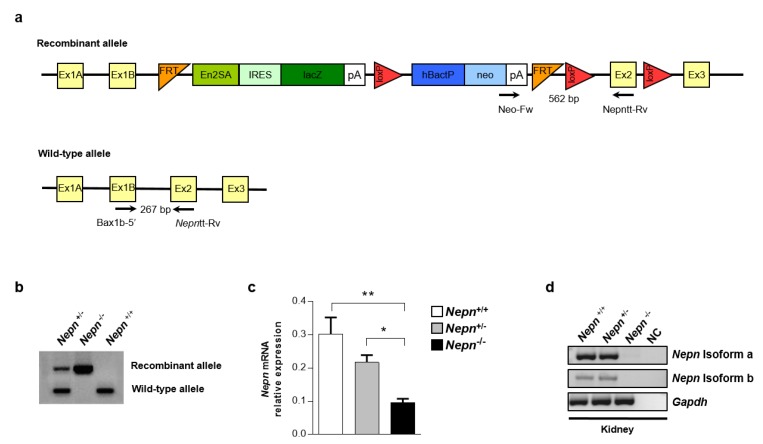
Generation of a mouse model for *Nepn* gene depletion. (**a**) Graphical representation of Nepn mutated locus in mESCs used to generate *Nepn* KO first mouse Primer designing for RT-PCR are indicated as arrows in the picture. (**b**) PCR analysis of genomic DNA isolated from mouse ear. The upper band in the panel displays the *Nepn* recombinant allele, while the lower band displays *Nepn* wild-type allele. Thus, the appearance of the upper band alone displays mutated homozygous allele; the lower band alone represents wild-type *Nepn* allele; while both bands together mean that the mouse is *Nepn* heterozygous. (**c**) RT-qPCR was performed to quantify the minimal expression of *Nepn* in *Nepn*^−/−^ mouse and relative controls. The data reported are normalized on *Gapdh* expression. Three replicates for each experimental point were performed. Error bars represent the standard deviation of normalized values (* *p* < 0.05, ** *p* < 0.001). (**d**) *Nepn* isoforms a and b expression in *Nepn*^−/−^ mouse kidney and relative controls. Full blot is showed in Supplementary Information.

To shed light on *Nepn* role in pancreatic development, mESCs from Komp Repository, carrying a genetically modified *Nepn* allele (Figure 3a), were microinjected into C57BL/6N blastocysts and transferred to a pseudopregnant recipient mice [20]. Chimeric offspring were then mated with wild-type mice which resulted in a certain percent of heterozygous progeny carrying the transgene in F1. Finally, the heterozygous mice were crossed to obtain homozygous mutant mice in F2. To discriminate homozygous from heterozygous mice, a genotyping analysis was performed using two different forward primers, each one specific for the wild-type or recombinant allele, in combination with a common reverse primer (Figure 3a), which resolved in a different amplicons size (Figure 3b). To avoid the problem of neo toxicity in homozygous *Nepn*^KOfirst^ mouse, neo expression cassette was removed by crossing muted mouse with C57BL/6N congenic constitutive Cre deleted mice [21]. All experiments illustated are peformed using a *Nepn*^KOfirstΔneo^ mice (so called *Nepn*^−/−^ or *Nepn*^+/−^). Analyses of Mendelian ratio from breeding of *Nepn* heterozygous mutant mice (Nepn^+/−^) show no altered Mendelian ratio suggesting that loss of *Nepn* does not affect mice survival (Table 1).

Moreover, WT and *Nepn* KO littermates were similar in size, in the appearance of the fur and gross morphology. In order to further validate the absence of both *Nepn* isoforms in knockout first mouse, we performed an RT-qPCR on the transcript’s portion shared by both isoforms. Since it was still possible to appreciate a residual expression of the gene in *Nepn*^−/−^ mouse (Figure 3c), we performed an RT-PCR to discriminate which of the two isoforms was still expressed. As shown in Figure 3d, it was possible to detect a slight expression of *Nepn* isoform a, whereas *Nepn* isoform b was completely shut down. We cannot rule out that this tiny expression could overcome KO affects.

### 2.4. Characterization of Pancreatic and Renal Functionality

**Figure 4:**
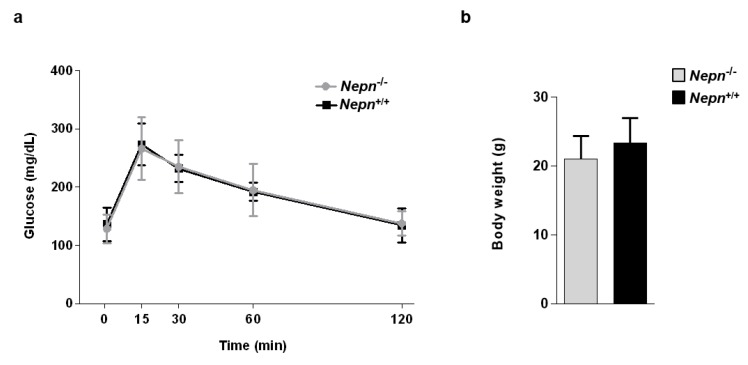
*Nepn* null mice have normal glucose regulatory function and body weight. (**a**) Intraperitoneal Glucose Tolerance Test (IPGTT). Evaluation of blood glucose of Nepn^−/−^ mice compared to *Nepn*^+/+^ in a group of 4–6 months old mice. Blood glucose was measured at the indicated time points after intraperitoneal glucose injections (*Nepn*^+/+^: seven mice; *Nepn*^−/−^: seven mice). The plasma glucose concentration peaked at 15 min after the glucose challenge and then gradually returned to normal level throughout the experiment—no significant differences between wild-type and *Nepn* null mice can be observed (*p* = 0.9, *n* = 7 for each group). Data are expressed as the mean ± SD. (**b**) The bodyweight of 4 and 6 months old mice were from wild-type and *Nepn* null groups. Results are means ± SD. T-test revealed no discernible differences between the genotypes (*p* = 0.26, *n* = 6 for each group).

Since our main interest is to characterize the role of *Nepn* in pancreatic homeostasis, we evaluated the pancreatic functionality of the *Nepn* knockout mouse model, performing an intraperitoneal glucose tolerance test (IPGTT) [22]. After intraperitoneal injection, we measured the blood glucose levels at different time points during the following 120h, and as shown in Figure 4a, *Nepn*^−/−^ animals have the same glucose induction and clearance curve compared to *Nepn*^+/+^ mice. This shows out that the absence of *Nepn* does not result in any type of alteration of glucose metabolism in mice 4–6 months old. The same measurements were carried out on mice at different ages (7–12 months), but still, no differences were observed (see Supplementary Figure S1). The intraperitoneal glucose administration can exclude the implication of a response driven by incretin hormones, which are gut peptides that are secreted after nutrient intake and stimulate insulin secretion together with hyperglycemia [23]. We, therefore, examined whether *Nepn* gene depletion may affect the body weight control at different ages. *Nepn*^−/−^ mice body weight was measured and compared to *Nepn*^+/+^ mice: both mice cohorts gained similar amounts of weight, indicating that the absence of *Nepn* is not directly correlated to metabolism and nutrient assimilation (Figure 4b and Supplementary Figure S1).

*Nepn* transcript is predominantly expressed in the kidney in adult mice [17], therefore we investigated the impact of *Nepn* deficiency on renal functionality. *Nepn*^−/−^ mice were accommodated in metabolic cages and urine volume, osmolality, electrolytes, and creatinine clearance were evaluated to assess renal homeostasis. Table 2 summarizes urinary parameter values measured in mice of 4–6 months old, showing the absence of significative variations between wild-type and mutant mice in renal functionality. The mice were monitored until 12 months, evaluating the same renal parameters to asses that no metabolism changes could occur in aged mice (Supplementary Table S1). Finally, serum analysis showed no differences in triglycerides, total cholesterol, urea, calcium and phosphorus levels (Supplementary Figure S2).

### 2.5. Generation of Nepn Deficient mESCs by RNA-Guided CRIPSR/Cas9

**Figure 5:**
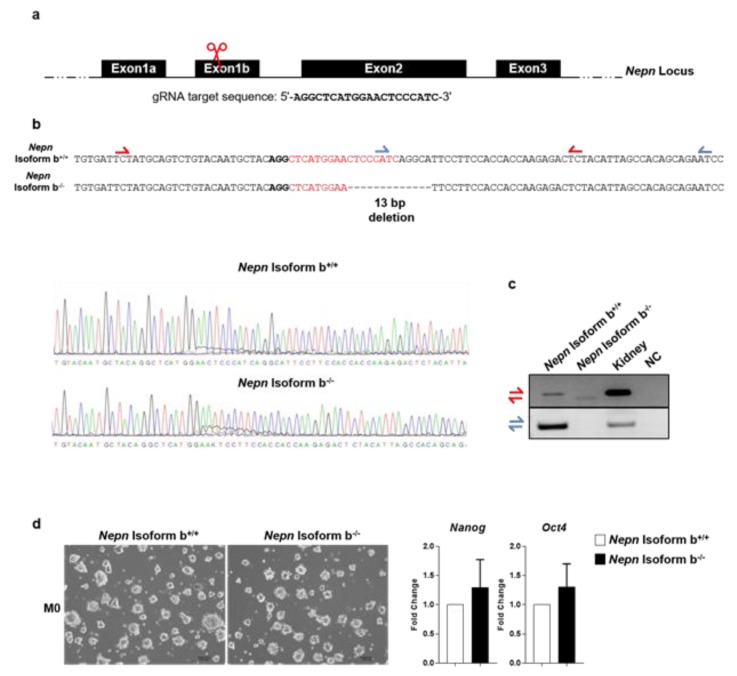
Generation of *Nepn* knockout mESCs. (**a**) Schematic diagram of the location and sequence of gRNA designed to target Exon1b of *Nepn* gene. (**b**) Chromatogram of the representative wild-type and CRISPR/Cas9 *Nepn* mutant clone. The interpretation shows mutated allele aligned against the wild type sequence. The bold letters represents the PAM sequence, while the dotted lines indicate deletions. Red and blue half arrows indicate the primer pairs used for the validation of CRISPR/Cas9 deletion. (**c**) Identification of a deletion in *Nepn* Isoform b gene sequence. Two PCR were performed once using primers flanking the gRNA target region, which leads to a smaller amplicon in the mutated allele. The second PCR is performed using a forward primer that anneals on the deleted region, resulting in no amplification on the mutated allele. (**d**) (Left) Representative brightfield images of undifferentiated (M0) wild type mESCs, and *Nepn* Isoform b knockout mESCs colonies. The colonies look alike and cells do not present any differences in generating colonies. Scale bars: 100 μm. (Right) RT-qPCR results show the expression level of stem cell markers (*Oct4, Nanog*)—no significant differences between wild type mESCs and *Nepn* Isoform b knockout mESCs can be observed. Full blot is showed in Supplementary Information.

Since *Nepn*^−/−^ mouse has shown no phenotype, we moved to a new cell system to pinpoint *Nepn* role in endodermal development, generating a homozygous deletion for *Nepn* in mESCs using CRISPR/Cas9 genome editing technology. First, we designed a gRNA to target *Nepn* isoform b (Figure 5a), which was found to be the exclusive one expressed in mouse development (Figure 2a). Second, the guide RNA was inserted in a construct encoding Cas9 for homologous recombination in Rosa26 genomic locus and then electroporated into wild-type mESCs. Finally, after selection, resistant clones were picked and expanded. Sanger-sequencing results revealed, among all picked colonies, a specific clone carrying a biallelic 13 bp deletion in *Nepn* exon1b (Figure 5b). The biallelic deletion causes a frameshift mutation few bases downstream ATG, resulting in a truncated peptide of 90 amino acids (Supplementary Figure S3). To further confirm the biallelic deletion, the selected clone was tested by RT-PCR using two different primer pairs. The first pair flanks the deleted region, which resolves in a band shift due to the different length in the amplicon size (Figure 5c top); the second pair has the forward primer which anneals on the deleted fragment leading to an amplification product only in the wild-type cell line (Figure 5d bottom). *Nepn* mutant mESCs colonies were indistinguishable in size and shape from unmodified cells (Figure 5d left), and growth rate was found to be comparable (data not shown). Moreover, we observed that the *Nepn* isoform b^−/−^ mESCs showed no apparent loss of pluripotency since the expression of *Oct4* and *Nanog* was not reduced (Figure 5 right), indicating that neither the genetic modification nor the absence of *Nepn* affected pluripotency and self-renewal properties.

### 2.6. Nepn Decifiency Impairs Endoderm Lineage Commitment

**Figure 6:**
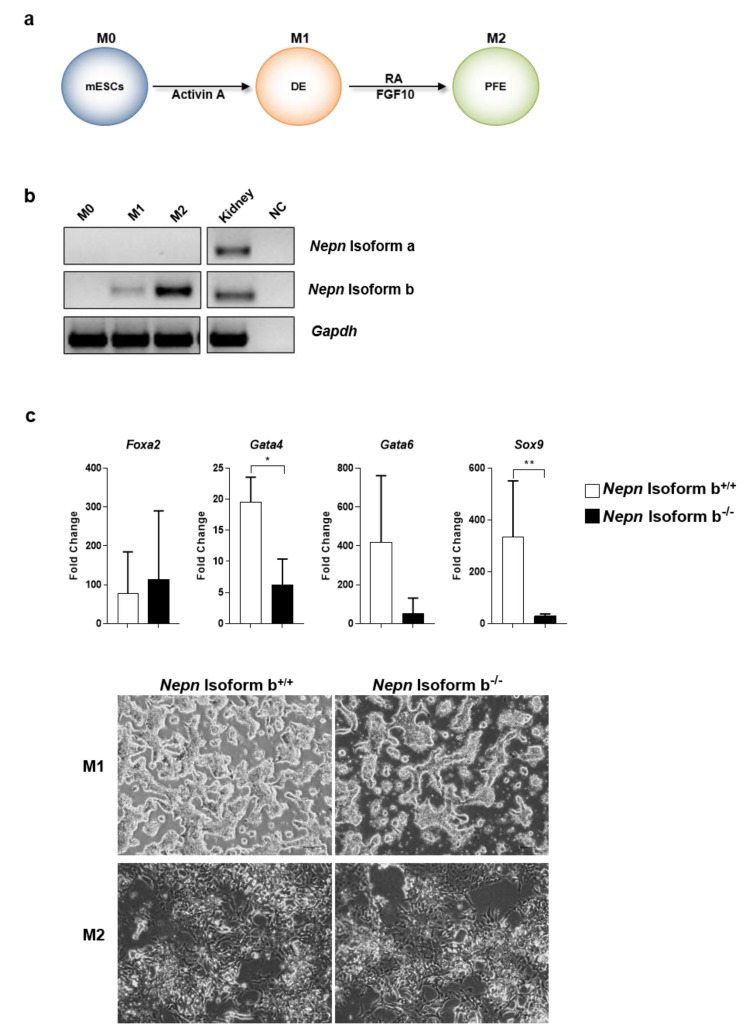
Evaluation on *Nepn* decifiency during endoderm differentiation (**a**) Schematic protocol of directed differentiation from mESCs into posterior foregut endoderm (PFE). ESCs were plated at 50,000 cells/cm^2^ at day0 (M0) in a pro differentiative medium supplemented with Matrigel (200 μg/mL) and Activin A (20 ng/mL) to induce the DE formation (M1). The cells were then treated with Retinoic Acid (5 μM) and FGF10 (10 ng/mL) until the end of protocol to promote the PFE formation (M2). (**b**) *Nepn* isoforms expression through the in vitro differentiation was evaluated by RT-PCR. Kidney cDNA was used as a positive control (Ctrl). (**c**) (Top) RT-qPCR analysis of the DE marker (*FoxA2*) and PFE markers (*Gata4, Gata6* and *Sox9*). The mRNA levels were normalized to *Gapdh* expression and reported as fold change with respect to the value in M1. Values shown are mean ± SD, based on triplicate assays. Statistical analyses were performed using Student’s t-test, where *p* < 0.05 was considered significant. (* *p* < 0.05, ** *p* < 0.01). (Bottom) Cells morphology during in vitro endoderm differentiation. Representative brightfield images of wild type and *Nepn* Isoform b knockout differentiated cells at M1 and M2. Images were taken with a Leica DMi8 at 10× magnification. Scale bar: 100 µm. Full blot is showed in Supplementary Information.

In vivo *Nepn* expression is restricted to a small subpopulation of endodermal cells at E7.25; afterwards (E8.0–8.5), this was found to be expressed in a wider region known as the midgut [18], to then relocate to the limited region of DPB [15]. We, therefore, focused on the possibility that *Nepn* may play a role in endodermal development and used an in vitro differentiation protocol, previously established in our laboratory, which is intended to recapitulate the endoderm development in vivo. Briefly, definitive endoderm (DE) formation (Metastate 1, M1) is induced during the priming step by Activin A. Sequently, the cells are further directed toward posterior foregut endoderm (PFE) (Metastate 2, M2) by RA and FGF10 (Figure 6a). We also confirmed that, as in vivo also in vitro, *Nepn* isoform b is the exclusive isoform expressed through developmental stages (Figure 6b). Finally, we induced mutant and wild-type cells to differentiate, and evaluated the expression of lineage specific markers. RT-qPCR analyses showed that the expression of DE marker *FoxA2* remained mainly unchanged in *Nepn* isoform b^−/−^ compared to *Nepn* isoform b ^+/+^ suggesting that definitive endoderm induction was not affected by biallelic mutation of *Nepn* (Figure 6c top). Interestingly, although there was no visible morphology difference between wild type and mutant cell line (Figure 6c bottom), the absence of *Nepn* isoform b appeared to affect formation of PFE, since its specific markers, *Sox9*, *Gata-4* and *Gata-6*, turned out to be expressed to a lesser extent in mutant cell line compared to wild-type (Figure 6c top).

## 3. Discussion

The embryonic definitive endoderm (DE) gives rise to organs of the respiratory and gastrointestinal tract including the pancreas, liver and epithelia of the colon and lung. Due to poor knowledge of molecular mechanisms that guide the in vivo endoderm specification, interest in the identification of novel endoderm genes is growing. On the other hand, elucidation of cellular components and genes governing tissues development is critical to understand the cause of organ disorders and cancers and will lead to novel therapies for tissue and organ regeneration. *Nepn* is a member of small leucine-repeat (SLRP) family of proteins. It is a secreted, N-glycosylated inhibitor of TGF-β and is expressed in the early stages of murine endoderm specification (E7.5–11.5) [15,17,18]. Here, a novel transcript of mouse *Nepn* gene was identified through bioinformatics tools and molecular biology techniques. This novel transcript, named *Nepn* isoform a, was found to be expressed in different tissues, but not during embryo development. The alignment of isoforms protein sequences highlighted the absence of the secretion signal peptide at the N-terminus. Since cellular localization of a protein plays an important role in defining its function, we speculate that the absence of important domains in the smaller variant might directly affect the secretion capability and activity of this protein. To further characterize *Nepn* isoform b role in endoderm patterning, we generated a mouse model with genetic depletion of *Nepn* isoform b, but found no obvious phenotype alterations, not even pancreas and renal functionality. One possible explanation for this apparent lack of an effect could be the presence of residual expression of *Nepn* isoform a, which could overcome KO effects. Moreover, it is possible that *Nepn* isoform b function may instead rely on other SLRP family members, since, e.g., Decorin (DCN) has been shown to bind TGF-β as a regulator of growth factors functions [24,25,26]. At present, 15 SLRP members have been cloned and partially characterized, and most are located in clusters on mouse and human chromosome [27]. Here, we have also generated a nullizygous *Nepn* isoform b knock-out mESC line using CRISPR/Cas9 system. This technology, due to its advantages in the high efficiency and specificity of gene targeting, is largely used in gene-editing [19]. Combining embryonic stem cell technology with CRISPR/Cas9 gene-editing provides an important strategy to clarify the biological role of markers, the understanding of normal embryonic development as well as the pathogenesis of diseases. We applied an in vitro differentiation protocol, previously established in our laboratory [15], which allows for robust and efficient generation of endodermal cells within eight days under chemically defined conditions. Interestingly, it is notable that posterior foregut markers, such as *Sox9*, *Gata-4* and *Gata-6*, turned out to be expressed to a lesser extent in mutant cell line compared to wild-type. It has been reported that the homozygous deletion of both *Gata4* and *Gata6* is necessary to disrupt pancreas development in mice [28,29,30]. Moreover, Xuan et al. recently demonstrated the importance of GATA4/6-mediated inhibition of hedgehog signaling as a major mechanism regulating pancreatic endoderm specification during patterning of the gut tube [31]. Our findings suggest that *Nepn* isoform b KO, interfering with *Gata4* and *Gata6* expression, may have an effect on the posterior foregut development and on the cell ability to reach posterior foregut metastate. Thus, it will be interesting to characterize the molecular interaction between *Nepn* isoform b-*Gatas* signaling during mouse endoderm development. Moreover, *Nepn* isoform b is expressed in adult mouse tissues, derived from the other two germ layers. We, therefore, speculate that it could also play a role in the specification of mesoderm and ectoderm derived tissues. Hence, it will be fascinating to explore *Nepn* isoform b function in differentiation assays towards the other two germ layers.

## 4. Materials and Methods

### 4.1. Ethics Statements and Animal Experiments

All animal studies were conducted at Biogem S.c.a.r.l. Ariano Irpino, AV (Italy), Preclinical Research and Development Service. Animals have been housed and used following the rules of the Italian laws (DL.vo N° 116—27/01/1992 and related) and of the EU directive (2010/63/UE—22/09/2010) on the protection of animals used for experimental purposes. All the in vivo procedures were in compliance with the *Guide for the Care and Use of Laboratory Animals* (United States National Research Council, 1996). All the in vivo experimental activities were evaluated and approved by the Committee for the Ethics of the Experimentations on Animals (CESA) and by Organismo Benessere Animale (OBA) of Biogem with ID: 7717 (Protocol Number: 7F782_20; 8 May 2017) and were authorized by the Italian Minister of Health (Authorization Number 384/2017-PR) according to the decree nr 100/2006 and later decree 26/2014. Wild type C57BL/6J (herein referred to as B6) mice were purchased from Charles River Laboratories (Wilmington, MA, USA). Animals were housed in an animal house under controlled conditions of temperature (22 ± 1 °C), humidity (55 ± 10%) and lighting on a 12-h light/12-h dark cycle and were supplied with standard rodent food and water ad libitum. Mice for testing were produced by crossing heterozygous females with heterozygous males. Littermate controls were used for all experiments. For tissue collection, all surgery was performed under anesthesia. All efforts were made to minimize suffering.

### 4.2. Generation of a Constitutive Nepn Knockout Mouse Line

For the generation of the *Nepn* KO first mouse line, one positive ES cell JM8.N4 clones (EPD0686_5_C01), containing a *Nepn* modified locus (Nepn knockout first allele), were purchased from Knockout Mouse Project (KOMP) Repository. Briefly, this knockout first allele contained a 2080 bp 5′ homology arm, an FRT-flanked cassette containing a *lacZ* reporter gene driven by *Nepn* promoter, a PGK neomycin resistance gene for positive selection (both separated by a loxP sequence) and two loxP sites flanking exons 2 and 3 of *Nepn* gene as well as a 6930 bp 3′ homology arm. To confirm the presence of the second loxP site flanking the exons 2 and 3 in these ES clones, long-range PCR was used to amplify the 3′ modified homology arm using KOMP primers. The presence of the loxP site generates a new restriction site for the restriction endonuclease SacI. Thus, digestion with this enzyme and sequence analysis allowed us to confirm the presence of this modification, and therefore, the presence of the *Nepn* modified locus. The positive JM8.N4 modified clone containing *Nepn* knockout first alleles was subsequently injected into C57Bl/6N blastocysts. Only male mice positive for germ-line transmission were used as the founder of the *Nepn* knockout first mouse line. By breeding *Nepn* heterozygous mutant mice, we obtained the constitutive *Nepn* knockout mice. Mouse genotyping was performed with genomic DNA samples isolated from ear. Standard PCR protocols were used to amplify the wild-type and knockout first alleles of the *Nepn* gene. Mice genotyping was verified by PCR using the following primers: Cds-Neo, Bax1b-5′ and Cds-*Nepn*-tt (Supplementary Table S2). To remove neo expression cassette, *Nepn* KO first mice were intercrossed with C57BL/6N congenic constitutive Cre deleted mice (Tang SH, Silva FJ, Tsark WM, Mann JR, 2002) and additional rounds of breeding were then performed in order to produce animals that were negative for the Cre transgene. Mouse genotyping was performed with genomic DNA samples isolated from ear biopsies that were taken from each animal at weaning. Standard PCR protocols were used to amplify the wildtype and knockout alleles of the *Nepn* gene. PCR was performed using the following primers: *lacZ*-(Cre)-Fw, Int1-Fw and Int1-Rv.

### 4.3. Cell Culture and Differentiation Protocol

Undifferentiated wild-type and the CRISPR/Cas9 knockout mESCs were cultured on gelatin-coated feeder-free plates in Dulbecco’s Modified Eagle Medium (DMEM, Sigma Aldrich, St. Louis, MO, USA) supplemented with 15% FBS (HyClone, Logan, UT, USA), 1000 units/mL ESGRO^®^ leukaemia inhibitory factor (LIF) (Merck Millipore, Burlington, MA, USA), 1.0 mM sodium pyruvate (Invitrogen, Waltham, MA, USA), 0.1 mM non-essential amino acids (Invitrogen), 2.0 mM L-glutamine (Invitrogen), 0.1 mM β-mercaptoethanol (Sigma Aldrich) and 500 U mL^−1^ penicillin/streptomycin (Invitrogen). ESCs were incubated at 37 °C in 5% CO2; medium was changed daily and cells were split every 2 to 3 days routinely. For the endoderm differentiation, 5 × 10^4^/plate cells were seeded in 35 mm dishes, as a feeder free monolayer. The differentiation medium consists of DMEM Low Glucose (Lonza, Valais, Switzerland), supplemented with 5% FBS (HyClone), 2 mM L-Glutamine (Gibco), 1 mM non-essential amino acids (Gibco, Dublin, Ireland), 100 U-μg/mL Penicillin/Streptomycin (Gibco), 0.1 mM β-Mercaptoethanol (Sigma Aldrich,, depleted of LIF, with 200 μg/mL Matrigel (BD Biosciences, Qume Drive, San Jose, CA, USA) and treated sequentially with several molecules at different time point for 8 days. These factors included activin A (20 ng/mL, R&D Systems, Minneapolis, MN, USA), all trans retinoic acid (RA, 5 μM, Sigma Aldrich), fibroblast growth factor 10 (Fgf10, 10 ng/mL, R&D Systems), cyclopamine (CYC, 10 μM, Sigma Aldrich) and N-N-(3,5-difluorophenacetyl)-Lalanylsphenylglycinet- butylesterm (DAPT, 5 μM, Sigma Aldrich). Medium was changed every 2 days.

### 4.4. Vector Construction

SpCas9 was used for editing the first exon of the *Nepn* gene. The mammalian codon-optimized Streptococcus pyogenes Cas9 gene was obtained from VP12 plasmid (Addgene plasmid ID 72247). It was digested with the two restriction enzymes *NotI* and *PmeI* and cloned into pENTR1A no ccDB (w48-1) plasmid (Addgene plasmid ID:17398). The gRNA expression cassette was amplified from the BPK1520 plasmid (Addgene plasmid ID: 65777), the amplicon was digested with *SpeI* and cloned into the pENTR1A no ccDB/Cas9 vector. gRNA4 was designed using crispr.mit.edu web tool. Paired oligos corresponding to *Nepn* gRNA4 (5′-AGGCTCATGGAACTCCCATC-3′) were cloned into the vector. In vitro recombination between pENTR1A no ccDB/BPK1520/Cas9 and pBS-Rosa26/ccDB vectors was obtained using the Gateway LR Clonase II enzyme mix according to the manufacturer’s directions (Invitrogen).

### 4.5. CRISPR/Cas9 Genome Editing

pENTR1A no ccDB/BPK1520/Cas9-pBS-Rosa26/ccDB construct was electroporated into mESCs. One to two weeks later, colonies were growing in the culture plate. Using 100 μL pipette tips, the colonies were picked up and placed individually in the 96 well culture plate filled with trypsin. After dissociation into single cells, the colonies were transferred onto gelatin-coated 24-well plates and then expanded in 10 cm plates separately. Furthermore, the genotyping by PCR/TA-cloning and chromatogram sequencing were used to analyze the mutations and select the positive clones for analysis and differentiation experiments. The primers (5′–3′) used for genotyping are *Nepn* Crispr F3-R2 (see Table S2).

### 4.6. RNA Extraction, RT-PCR Analysis and Quantitative Real Time RT–qPCR

Total RNA was extracted using TRIzol reagent (Invitrogen) and cDNA was synthesized using iScript cDNA Synthesis kit according to the manufacturer’s instructions (Bio-Rad, Hercules, CA, USA). 1 μg of total RNA was used for each cDNA synthesis. Primer 3 software (http://primer3.ut.ee/) was used to design the oligo primers setting the annealing temperature to 59–61 °C for all primer pairs. Oligo sequences are reported in Table S2. Each PCR reaction was performed with 25 ng of single stranded cDNA as template and the appropriate set of forward and reverse primers. PCR amplification conditions are reported in Table S3 for *Nepn-201,* and *Nepn-202* was performed under the following: initial denaturation for 3 min at 95 °C; repeated 35 cycles of denaturation for 30 s at 93 °C; annealing for 20 s at 58 °C; extension for 40 s at 72 °C. This was followed by final extension at 72 °C for 8 min.

For gene expression analyses, the same amount of cDNA (25 ng) was used for each PCR reaction with each primer pair (forward/reverse primers mix: 0.2 μM, in a final volume of 25 μL). Real time-qPCR analysis was performed using the iTaq™ Universal SYBR^®^ Green Supermix (Bio-Rad, Hercules, CA, USA) in a 7500 Real-Time PCR System (Applied Biosystems, Foster City, CA, USA) under the following conditions: 2 min at 50 °C, 10 min at 95 °C, followed by 40 cycles of 15 s at 95 °C and 1 min at 60 °C. The *Gapdh* probe served as a control to normalize the data. The gene expression experiments were performed in triplicate on three independent experiments and a melting analysis was performed at the end of the PCR run. To calculate the relative expression levels we used the 2^−DDCT^ method [32,33].

### 4.7. Experimental Study for Renal Functionality

All experiments were conducted on age and gender-matched animals. Mice were housed individually in metabolic cages for 5 days at 23 °C with a 12 h dark/light cycle. Food and water were offered ad libitum. After 4 days of adjustment, physiological parameters were evaluated on day 5. 24 h urine output was collected under mineral oil to prevent evaporation. Urinary osmolality was measured by Osmometer 3320 (Advanced Instrument, Inc, Norwood, MA, USA)

### 4.8. Intraperitoneal Glucose Tollerance Test (IPGTT)

All experiments were conducted on age and gender-matched animals. Mice were housed individually in cages at 23 °C with a 12 h dark/light cycle and were fasted for 18 h. After fasting, mice were weighed and received an intraperitoneal injection of glucose (2 mg/g of body weight). Blood glucose levels were measured before glucose injection and after 15, 30, 60 and 120 min after glucose injection by using the OGCare glucometer (Biochemical Systems International S.r.l., Arezzo (AR), Italy).

### 4.9. Serum Analyses

Blood samples from mice at different ages (4–6 months, 7–12 months) were collected and centrifuged for 5 min at 5000 rpm. Serum supernatants were recovered and analyzed by SCIL VITROVET; in particular, triglycerides (mg/dl), total cholesterol (mg/dl), urea (mg/dl), calcium (mg/dl) and phosphorus (mg/dl) were measured.

### 4.10. Statistics

Graphpad Prism 5 software was used to perform statistical analysis. Values are presented as mean ± SD. *p*-values were determined using a two-tail unpaired t-test. *p* < 0.05 was used as a threshold for statistical discernibility.

Data Availability. The datasets generated during the current study are available from the corresponding author upon reasonable request.

## Figures and Tables

**Table 1:** Genotypes of the progeny from *Nepn* heterozygous mutant mice (*Nepn*^+/−^).

*n* = 86	*Nepn* ^+/+^	*Nepn* ^+/−^	*Nepn* ^−/−^	*p*-value
Observed frequency	29.1%	53.5%	17.4%	0.2
Expected frequency	25%	50%	25%	

**Table 2:** Experimental study of renal functionality. Evaluation of urinary parameters in 24 h urine output of *Nepn*^−/−^ mice compared to *Nepn*^+/+^ in a group of 4–6 months old mice.

Urinary Parameters	*Nepn* ^+/+^	*Nepn* ^−/−^
Urinary Volume (mL)	1.86 ± 0.37	1.63 ± 0.25
Creatinine excretion (µmol/g body weight)	0.24 ± 0.03	0.23 ± 0.01
Na^+^/creatinine	22.75 ± 4.16	27.92 ± 4.41
K^+^/creatinine	20.36 ± 3.95	18.14 ± 2.23
Cl^−^/creatinine	62.91 ± 6.43	72.34 ± 3.43
Creatinine clearence	113.16 ± 14.91	97.56 ± 11.33

## Supplementary Materials

The following are available online at https://www.mdpi.com/1422-0067/21/1/8/s1. Supplementary Figure S1: Pancreatic functionality. (**a**) Intraperitoneal Glucose Tolerance Test (IPGTT). Evaluation of blood glucose of *Nepn*^−/−^ mice compared to *Nepn*^+/+^ in a group of 7–12 months old mice. In the figure, blood glucose levels are represented at 0, 15, 30, 60 and 120 min from the glucose administration (*Nepn*^+/+^: 7 mice; *Nepn*^+/+^: 7 mice). (**b**) Evaluation of mouse body weight of *Nepn*^−/−^ compared to *Nepn*^+/+^ mice during time life in a group of 7–12 months old mice. Supplementary Figure S2: Serum levels parameters in a group of 4–6 months old mice. Triglycerides, total cholesterol, urea, calcium and phosphorus were measured—no significant differences between *Nepn*^+/+^ and *Nepn*^−/−^ mice can be observed. Supplementary Figure S3: In *Nepn*^−/−^ mESCs a frameshift mutation generates several stop codons (indicates as *), thus, inhibiting protein synthesis. Supplementary Table S1: Experimental study of renal functionality. Evaluation of urinary parameters in 24 h urine output of *Nepn*^−/−^ mice compared to *Nepn*^+/+^ in a group of 7–12 months old mice. Supplementary Table S2: RT-PCR and RT-qPCR primers (5′–3′). Supplementary Table S3: PCR amplification conditions.

## Abbreviations

B6	C57BL/6J
CESA	Comitato Etico per la Sperimentazione Animale
DE	Definitive endoderm
E7.5	Embryonic day 7.5
ESCs	Embryonic stem cells
mESCs	Mouse embryonic stem cells
PS	Primitive streak
EMT	Epithelial-to-mesenchymal transition
MET	Mesenchymal-to-epithelial transition
VE	Visceral endoderm
PFE	Posterior foregut endoderm
DPB	Dorsal pancreatic bud
SLRP	Small leucine-rich repeat protein
IPGTT	Intraperitoneal glucose tolerance test
KO	Knock-out
Nepn	Nephrocan
WT	Wild-type
CRISPR/Cas9	(CRISPR)/CRISPR-associated systems 9
UTR	Untranslated region
RT-PCR	Reverse transcriptase-polymerase chain reaction
RT-qPCR	Real Time-quantitative polymerase chain reaction
SD	Standard Deviation
FRT	Flip-recombinase targets
PGK	Phosphoglycerate kinase 1
PAM	Protospacer adjacent motif
