# Supplementary material for: Insight into Nephrocan Function in Mouse Endoderm Patterning
Source: Int J Mol Sci. 2019 Dec 18;21(1):8. doi: 10.3390/ijms21010008 (PMC6981620; doi:10.3390/ijms21010008)
Supplement: Supplementary file 1 [file ijms-21-00008-s001.pdf]

Supplementary Information

Article

Insight into *Nephrocan* Function in Mouse Endoderm Patterning

Martina Addeo [1,2], Silvia Buonaiuto [2], Ilaria Guerriero [1], Elena Amendola [2,3], Feliciano Visconte [4], Antonio Marino [2], Maria Teresa De Angelis [1], Filomena Russo [1], Luca Roberto [1], Pina Marotta [1], Nicola Antonino Russo[1], Anna Iervolino[1], Federica Amodio[1], Mario De Felice [3], Valeria Lucci [2,3,*,†] and Geppino Falco [1,2,3,*,†]

[1] Istituto di Ricerche Genetiche "G. Salvatore", Biogem s.c.ar.l, Ariano Irpino, 83031 Avellino, Italy; martina.addeo@gmail.com (M.A.); ilaria.guerriero.ig@gmail.com (I.G.); mariateresadeangelis211285@gmail.com (M.T.D.A.); filomena_russo1982@libero.it (F.R.); luca.roberto@biogem.it (L.R.); russonico@gmail.com (N.A.R.); pinamarotta82@gmail.com (P.M.); amodio.federica@yahoo.it (F.A.); anna.iervolino@biogem.it (A.I)

[2] Dipartimento di Biologia, Università degli Studi di Napoli "Federico II", 80126 Napoli, Italy; buonaiutosilvia@gmail.com (S.B.); antoniomarmail@gmail.com (A.M.);

[3] Istituto per l'Endocrinologia e l'Oncologia Sperimentale "G. Salvatore", CNR, 80131 Napoli, Italy; mario.defelice@unina.it (M.D.F.); elena.amendola@unina.it (E.A.)

[4] CEINGE Biotecnologie Avanzate s.c.a.r.l., 80131 Napoli, Italy; visconte@ceinge.unina.it (F.V.)

* Correspondence: valeria.lucci@unina.it (V.L.); geppino.falco@unina.it (G.F.); Tel.: +39-081-679083 (V.L.); +39-081-679092 (G.F.)

† These authors contributed equally to this work

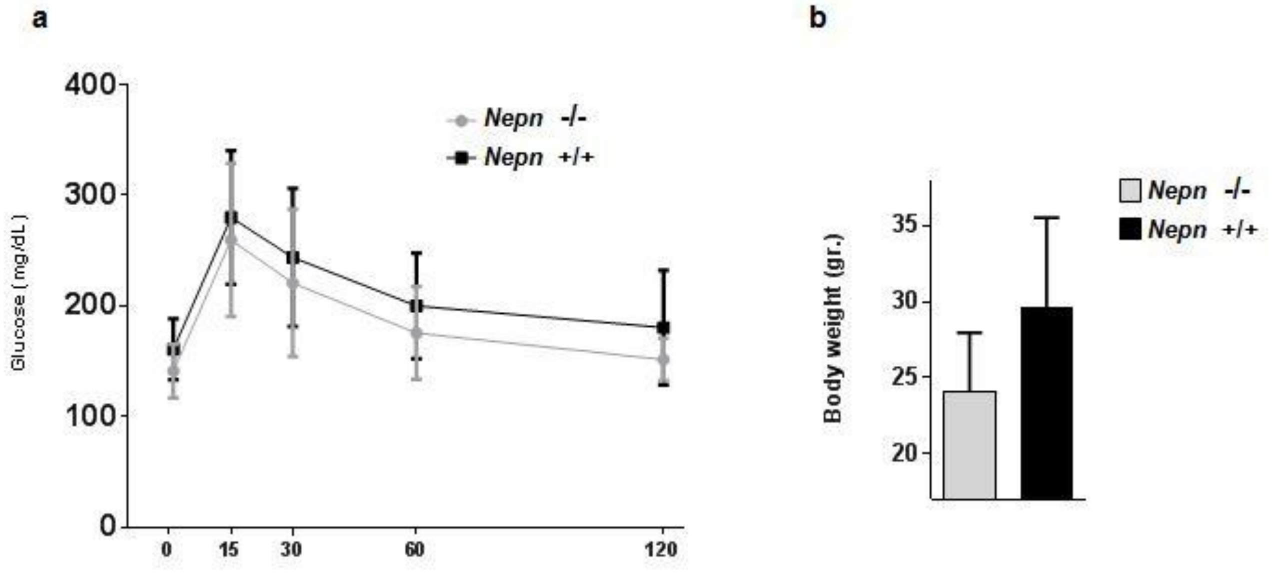

Supplementary Figure S1: Pancreatic functionality. **a**) Intraperitoneal Glucose Tolerance Test (IPGTT). Evaluation of blood glucose of *Nepn*$^{-/-}$ mice compared to *Nepn*$^{+/+}$ in a group of 7-12 months old mice. In figure, blood glucose levels are represented at 0, 15, 30, 60 and 120 minutes from the glucose administration (*Nepn*$^{+/+}$: 7 mice; *Nepn*$^{+/+}$: 7 mice). **b**) Evaluation of mouse body weight of *Nepn*$^{-/-}$ compared to *Nepn*$^{+/+}$ mice during time life in a group of 7-12 months old mice.

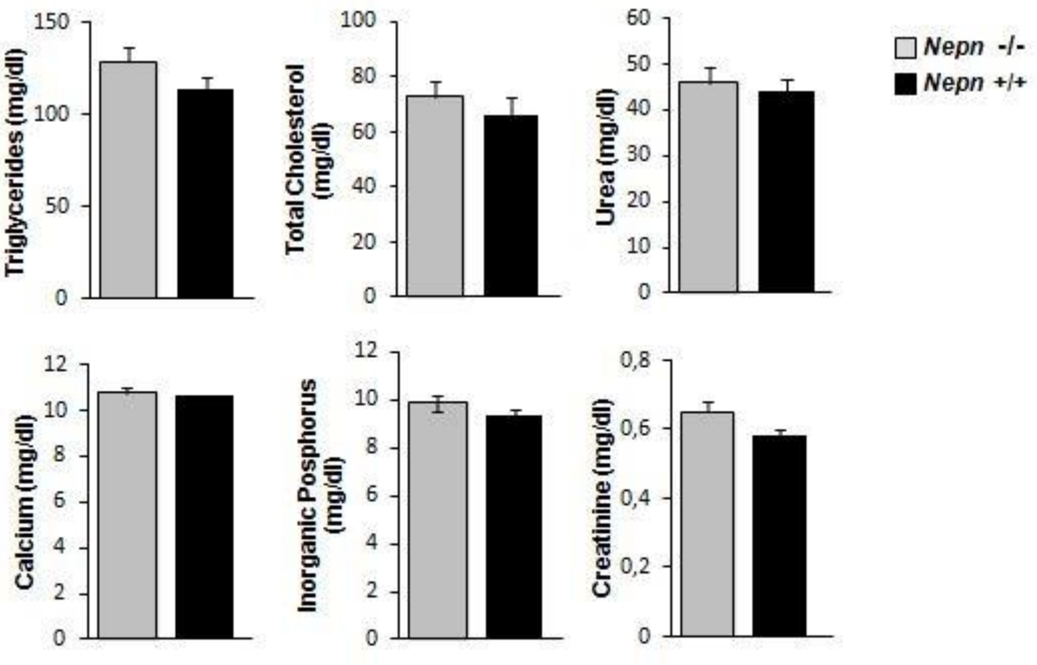

Supplementary Figure S2: Serum levels parameters in a group of 4-6 months old mice**.** Triglycerides, total cholesterol, urea, calcium and phosphorus were measured - no significant differences between *Nepn*$^{+/+}$ and *Nepn*$^{-/-}$ mice can be observed.

```
MHPLWAFLLG LSLTNGLSAN CPGRCSCDSM QSVQCYRLME FLPPPRDSTL ATAESSTFSY LTSLDCWLWK ILFCWPVEQS 80
LLKMTPSKL* VH*RL*NFGK IN*DKSPVLS QPTLRC*N*M IMPYVLYVDQ SLKD*RT*KS LNLKTT*SRP CLPACSPHLP 160
VCRV*WWMVT T*SPWLDL*A FRISST*AWR TTNSTLYQGM SSLPYRICSS SVSVVIF*LK FP*IYPNPCY L*RWRETSSK 240
WSGFAI*NTW RTCPIFTCQR TSCLPLMGHS S*PI*QLWRF PKTSFKCCLP GYHRGYRSLI VAVTSFRE*Q HQNSRTSET* 320
NICF*TTMLS ACSRLGPFRG VLSCPTWPWS RICCCLSL*G SLRPWPGWTS RAMPSRTWLK GSSGTSNNCR SSTSGTTGSR 400
P*ISRHWRGC LGSGISTWMG IPGTALAVSY EPGKS*RPRA RT*KGDSAQP QQSDRGRAGC PPRRS*GNVS ITYSRVRKAK 480
RPRRNPNPKT LPASDSTWMT TMTIMK*I 508
```

Supplementary Figure S3: In *Nepn*-/- mESCs a frameshift mutation generates several stop codons (indicates as *) thus inhibiting protein synthesis.

Supplementary Table S1: Experimental study of renal functionality. Evaluation of urinary parameters in 24h urine output of *Nepn*$^{-/-}$ mice compared to *Nepn*$^{+/+}$ in a group of 7-12 months old mice.

**Urinary Parameters**	***Nepn*** $^{+/+}$	***Nepn*** $^{-/-}$
Urinary Volume (ml)	2.14±0.78	2.36±0.69
Creatinine excretion (µmol/g body weight)	0.26±0.02	0.26±0.03
Na^+ / creatinine	27.42±2.78	24.01±3.15
K^+ / creatinine	27.92±2.91	34.66±4.40
Cl^- / creatinine	59.53±6.24	51.50±5.63
Creatinine clearence	120.59±8.83	113.65±15.73

Supplementary Table S2: RT-PCR and qRT-PCR primers (5’-3’

**Gene Name**	**Forward Primer 5′-3′**	**Reverse Primer 5′-3′**
Cxcr4	gtaaccaccacggctgtaga	agtagatggtgggcaggaag
Gapdh	cggagtcaacggatttggtcgtat	gaagatggtgatgggcttcc
Gata2	agctcatgactatggcagca	ccggttctgtccattcatct
Nanog	aaccagtggttgaagactagcaatggtc	ttccagatgcgttcaccagatagc
NeuroD	gctccagggttatgagatcg	ctctgcattcatggcttcaa
Oct4	ccgtgtgaggtggagtctggagac	cgccggttacagaaccatactcg
Foxa2	ctgggagccgtgaagatggaag	tccagcgcccacataggatg
Gata4	tcaaaccagaaaacggaagc	ctgctgtgcccatagtgaga
Gata6	caccatcacccgacctactc	gcatgcattgcacaggtaat
Sox9	ggtctgcctggactgtatgtggatg	ctgtccgatgtctctctgcaggag
Hnf6	caaagaggtggcgcagcgtatc	gctctttccgtttgcaggctg
Nkx6.1	gacagcaaatcttcgccctgg	ttctccgaagtccccttgagcc
Rex1	cagaagaaagcaggatcgcctc	gccacttgtctttgccgttttc
Nepn Iso B	gatgcagttgtgattctatgc	tagactctgttccactggc
Nepn Iso B (2)	aacctctgtgttggacaatgc	gtgctgcaaggcgattaagt
Nepn Iso A	agatccttctctgcaatgtgaac	tcttccagagccaacagtcc
Nepn Iso A (2)	agatccttctctgcaatgtgaac	gtgctgcaaggcgattaagt
*Nepn*_b FR	tgtgattctatgcagtctgtaca	ggattctgctgtggctaatg
Nepn Crispr F3-R2	aaaagattctgttatcagactccg	cgagggtcctctggatatggaagc
Nepn Crispr Del	tcatggaactcccatcaggc	tagactctgttccactggc
Nepn Crispr Ko	ctcatggaattccttccacc	tagactctgttccactggc
Cds-Neo	gggatctcatgctggagttcttcg	agaattccccttcacatctgcctcc
Bax1b-5'	tggtgagacatttgtagatt	agaattccccttcacatctgcctcc
lacZ	gggatctcatgctggagttcttcg	agaattccccttcacatctgcctcc

lacZ-(Cre)	cgtcagtatcggcggaattcc	tccccttcacatctgcctcc
Int1	gaaacttttcacagtatgg	tccccttcacatctgcctcc

Supplementary Table S3: PCR condition used in the study

Genes	Denaturation (temp./duration)	Cyclic Denaturation, Annealing and Elongation (temp./duration)	No. of Cycles	Final Elongation and Cooling (temp./duration)
Nepn*-202**<br>Nepn*-201**<br>***Gapdh* (Fig. 1b)**	95°C/5 minutes	95 °C/30 seconds<br>58 °C/20 seconds<br>72 °C/45 seconds	35	72 °C/5 minutes<br>4 °C/∞
Nepn* isoform a**<br>Nepn* isoform b**<br>***Gapdh* (Fig. 2b, 3d, 6b)**	95°C/5 minutes	95 °C/15 seconds<br>55 °C/15 seconds<br>72 °C/20 seconds	38	72 °C/5 minutes<br>4 °C/∞
**Recombinant allele**<br>**Wild-type allele (Fig. 3b)**	95°C/5 minutes	95 °C/15 seconds<br>60 °C/30 seconds<br>72 °C/45 seconds	10<br>(-1°C/cycles)	72 °C/5 minutes<br>4 °C/∞
		95 °C/15 seconds<br>50 °C/30 seconds<br>72 °C/45 seconds	30	72 °C/5 minutes<br>4 °C/∞
Nepn* isoform b**<br>Nepn* isoform b**<br>**(Fig. 6b)**	95°C/5 minutes	95°C/30 seconds<br>58°C/15 seconds<br>72°C/15 seconds	38	72 °C/5 minutes<br>4 °C/∞

Supplementary Table S2: RT-PCR and qRT-PCR primers (5’-3’)

Full gel image for Fig. 1b

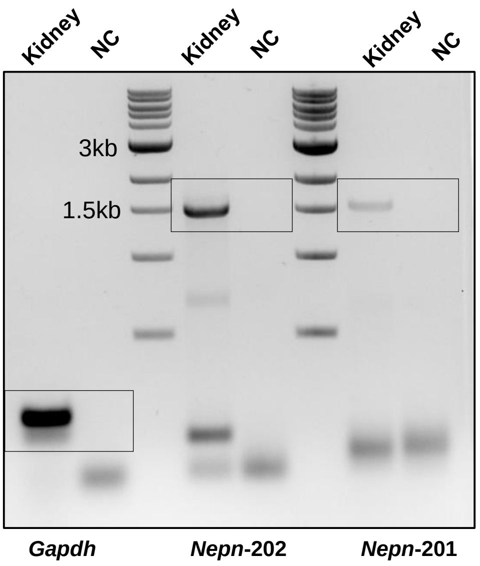

Full gel images for Fig. 2a

NA=Not Applicable

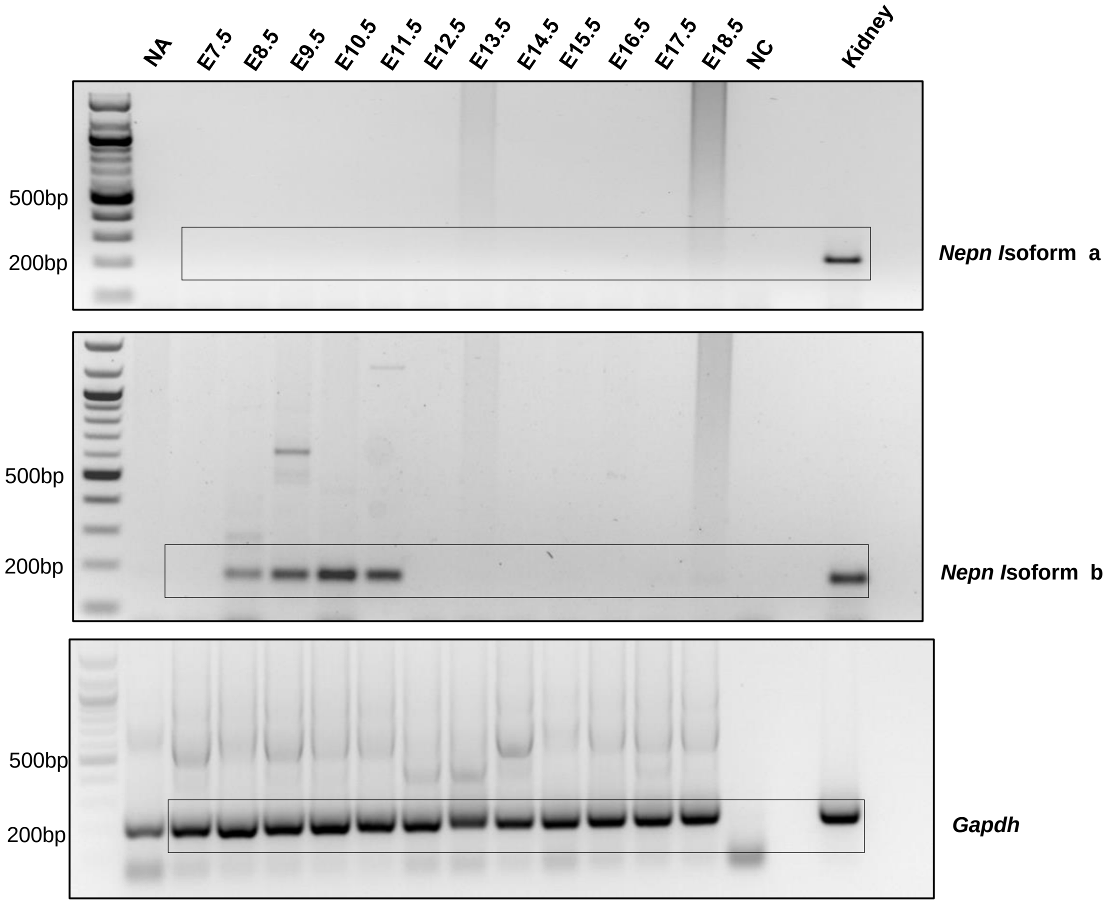

Full gel images for Fig. 2b

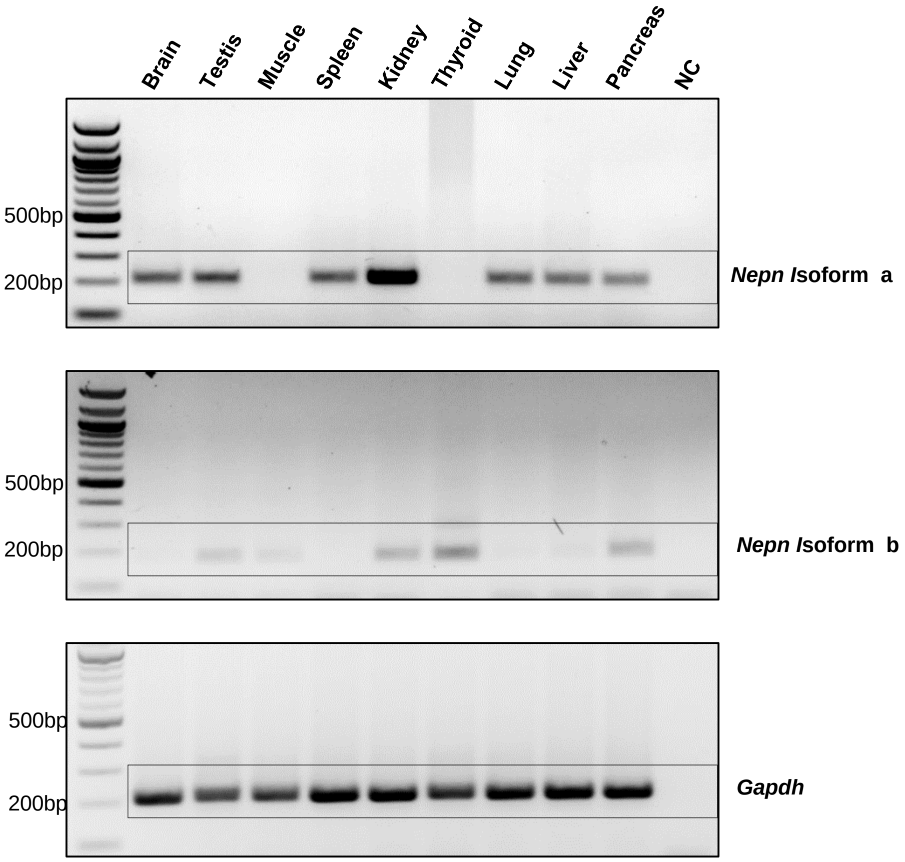

Full gel images for Fig. 3d

NA=Not Applicable

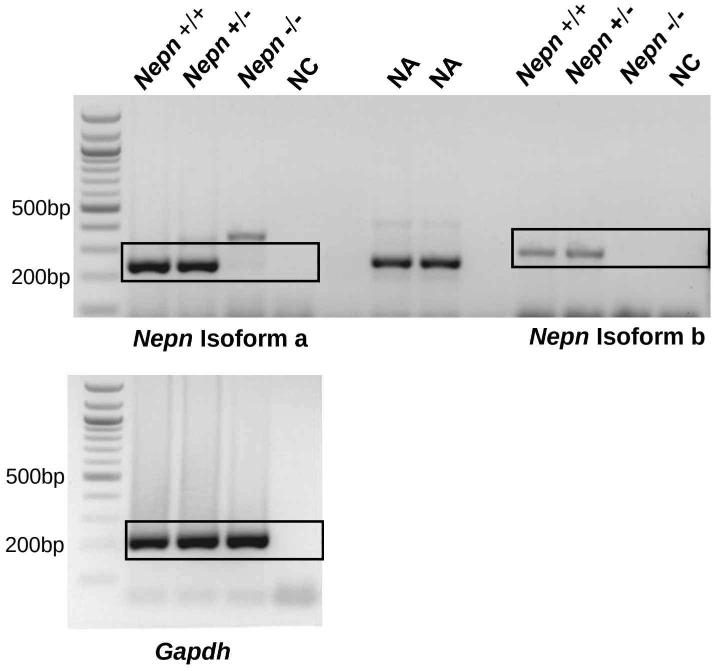

Full gel images for Fig. 5c

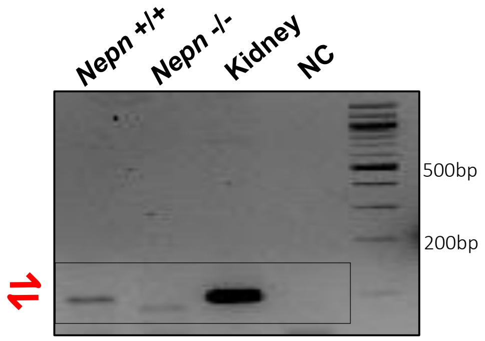

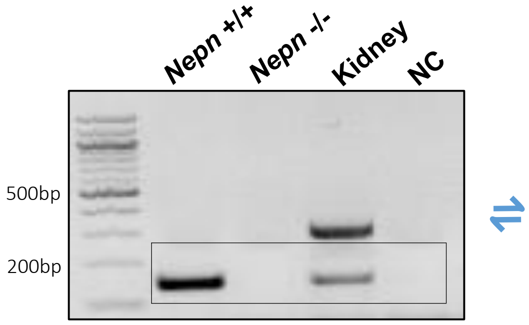

Full gel images for Fig. 6b

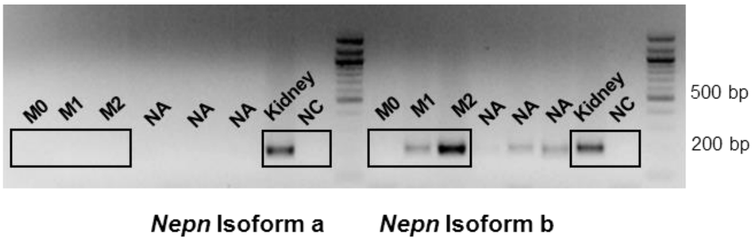

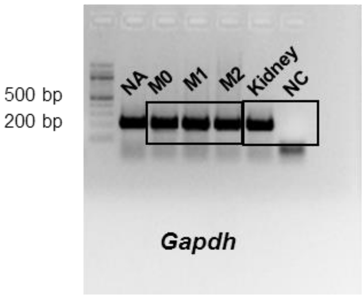

NA=Not Applicable
